# Modified Pedicle Subtraction Osteotomy for Osteoporotic Vertebral Compression Fractures

**DOI:** 10.1111/os.12589

**Published:** 2020-02-27

**Authors:** Sung‐Kyu Kim, Jae‐Yoon Chung, Yong‐Jin Park, Seung‐Won Choi, Hyoung‐Yeon Seo

**Affiliations:** ^1^ Department of Orthopedic Surgery Chonnam National University Hospital Gwangju Korea; ^2^ Department of Orthopedic Surgery Chonnam National University Medical School Gwangju Korea

**Keywords:** Kyphosis, Osteoporosis, Osteotomy, Spine fracture

## Abstract

**Objective:**

To evaluate the cases of osteoporotic compression fracture with kyphotic deformity treated with modified pedicle subtraction osteotomy and analyze the usefulness of this osteotomy.

**Methods:**

Twenty patients (nine men, 11 women; mean age, 66.4 years; mean follow‐up duration, 39.6 months) who underwent modified pedicle subtraction osteotomy at our hospital between November 2003 and July 2012 with a minimum follow‐up period of 2 years were included. All cases were injuries induced by a fall incurred while standing or lifting heavy objects without high‐energy trauma. The mean bone mineral density‐dual energy x‐ray absorptiometry (BMD‐DEXA) was 0.673 g/cm^2^ (0.571–0.740 g/cm^2^), which was ‐2SD below normal, indicating severe osteoporosis. The indications for surgery included gait disturbance due to severe pain with pseudarthrosis, increased kyphotic angle, and progressive neurological symptoms. We evaluated Japanese Orthopaedic Association scores, Oswestry Disability Index scores, bone union, change in kyphotic angle, estimated blood loss, complications, and the patient's overall clinical satisfaction.

**Results:**

The collapsed vertebrae were thoracic (T_9, 11, 12_) in 13 cases and lumbar (L_1_) in seven cases. The mean pre‐ and post‐operative kyphotic angles were 26.2 ± 9.9 degrees and 8.3 ± 8.1 degrees, showing an 18.3‐degree correction. Plain radiography at the last follow‐up showed a mean angle of 17.9 ± 12.6 degrees, indicating a 9.5‐degree correction loss. The overall correction was 8.2 degrees. Bony union was achieved at the last follow‐up in all cases. Clinical outcomes showed improvement in mean Japanese Orthopaedic Association score from 13.2 to 21.7 and mean Oswestry Disability Index score from 40.3 to 13.6. Overall clinical patient satisfaction showed 12 excellent results, five moderate results, and three poor results. Mean operation time was 4.2 h (range, 4–6 h). The overall mean estimated blood loss (EBL) was 1098 mL (range, 750–1370 mL). The mean hospital stay was 3.6 weeks (range, 2–6 weeks). There was one case of cauda equina syndrome at 2 days postoperatively, and two cases of screw loosening. There were no cases of distal junctional kyphosis, but there were two cases of proximal junctional kyphosis.

**Conclusions:**

Despite limited correction of kyphotic angle, our modified pedicle subtraction osteotomy technique resulted in satisfactory outcomes in our patients, who had persistent severe back pain, increased kyphotic angle with pseudarthrosis, and progressive neurologic deficits. Nevertheless, this procedure requires careful follow‐up and strict surgical indications because of the risk of neurological damage and technical problems.

## Introduction

With the aging population increasing, the incidence of osteoporosis is very high and osteoporotic vertebral compression fracture (OVCF) also increases with age. Especially, OVCF occurs in over 40% of individuals over 80 years of age, and in 25% of all postmenopausal women^1^. OVCF can lead to an increase in the kyphotic angle and cause localized pain. Most patients receive conservative treatment because their symptoms can be treated without complications. However, despite sufficient conservative treatment, some patients suffer from severe pain and neurological symptoms due to deterioration of vertebral collapse and spinal instability. In these cases, surgery may be an appropriate treatment method. However, there are not many studies on surgical treatment for OVCF because this method has high complication rates for this issue; this is due to OVCF occurring predominantly in old age with poor general condition or several underlying disease, and internal fixation is difficult due to weak bone quality^2^, ^3^, ^4^. Nevertheless, due to the increasingly aging population, the incidence of OVCF has increased^5^. As a result, to overcome the limitations of conservative treatment, the importance of active treatments, such as surgery, has become an essential topic. In particular, severe pain can develop from pseudarthrosis of a fractured vertebral body, and severe neurological symptoms can develop from the posterior translation of fracture fragments into the spinal canal or severe posterior kyphosis^6^. In these cases, conservative treatment is ineffective and surgical treatment must be considered. The surgeon evaluates the degree of vertebral body collapse and kyphosis, existence of pseudarthrosis, age, and neurological symptoms of the patient and determines which surgical method – such as vertebroplasty, posterior decompression and fusion, anterior reconstruction after spondylectomy, and osteotomy – will be most effective. Vertebroplasty has the advantage of being relatively simple and non‐invasive but difficult to use in severe OVCF, so surgical treatment is considered for patients with significant progression of kyphotic deformity, progressive neurological symptoms, or severe pain and disability.

Previous literature mainly reported posterior decompression, posterior fusion, or anterior reconstruction as treatment methods of OVCF, while only a few studies reported spinal osteotomy to treat OVCF. Spinal osteotomy may decompress the spinal canal, restore the sagittal balance, and correct the kyphotic deformity. In cases of pseudarthrosis due to healing failure of vertebral body fractures with kyphosis, or in cases of neurological impairments such as spinal cord compression or spinal stenosis, spinal osteotomy is a viable treatment option. Accordingly, we undertook this study to evaluate the clinical and radiological outcomes, complications, and the effect of our modified pedicle subtraction osteotomy.

The purpose of this report was to: (i) evaluate patients with OVCF with kyphotic deformity who underwent our modified pedicle subtraction osteotomy; (ii) analyze the clinical and radiological outcomes and complications of this osteotomy; and (iii) find out the usefulness of our spinal osteotomy for OVCF.

## Methods

### *Inclusion and Exclusion Criteria*

The inclusion criteria for this study were: (i) patients diagnosed with OVCF; (ii) patients who experienced failed conservative treatment for at least 3 months; (iii) patients who underwent modified pedicle subtraction osteotomy at our hospital between November 2003 and July 2012; and (iv) patients with a follow‐up period of at least 2 years.

Exclusion criteria were: (i) patients with normal bone mineral density (BMD); (ii) patients with cases of secondary osteoporosis, such as endocrine system, gastrointestinal, and thyroid disease; (iii) patients with cases of pathologic fracture; and (iv) patients with cases of fracture due to high energy trauma.

**Figure 1:**
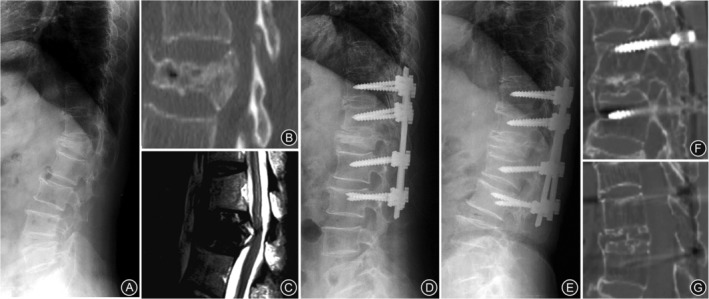
(A) Preoperative radiograph of a 71‐year‐old woman with L_1_ osteoporotic compression fracture accompanying functional paraplegia showing a kyphotic angle of 23.5 degrees. (B) A sagittal CT scan showing collapse of the vertebral body. (C) A sagittal T2WI magnetic resonance image showing cord compression. (D) Immediate postoperative radiograph showing that the kyphotic angle decreased to 10.4 degrees. (E) A radiograph taken at 5 years postoperative shows increased kyphosis to 27.3 degrees (16.9‐degree correction loss). However, the patient's pain relief and partial recovery of functional paraplegia were maintained. (F, G) CT scan taken at 5 years postoperative showing bone union of the osteotomy site and decompression of the spinal canal (F, intervertebral foramen area; G, central area).

The indications for surgery included gait disturbance by severe pain with pseudarthrosis, increased kyphotic angle, and progressive neurological symptoms (Fig. 1).

### *Patient Information*

There were twenty patients (11 women, nine men; mean age, 66 years [52–79 years]; mean follow‐up, 39.6 months [24–111 months]), with the individual demographic data shown in Table 1. In this study, no patients had a history of high‐energy trauma. Most cases were injuries induced by a fall incurred while standing or lifting heavy objects. We checked anteroposterior, lateral, flexion, and extension plain radiographs taken in the supine position and dual energy X‐ray absorptiometry (DEXA) to diagnose osteoporosis. In all cases, osteoporotic changes, such as increased radiolucency, cortical thinning, penciling of the vertebrae, and loss of trabeculation, were found on the plain radiograph^7^. The mean BMD‐DEXA was 0.673 g/cm^2^ (0.571–0.740 g/cm^2^), which was ‐2SD below normal, indicating severe osteoporosis. The collapsed vertebrae were thoracic (T_9, 11, 12_) in 13 cases and lumbar (L_1_) in seven cases. Collapse and kyphosis of the vertebral body were observed on the preoperative lateral plain radiographs of all patients. Intravertebral pseudarthrosis or instability was identified with clinical symptoms (pain, tenderness) and serial follow‐up plain radiographs as well as preoperative computerized tomography (CT) or magnetic resonance imaging (MRI).

**Table 1:** Demographic data

Patient	Age	Sex	Fracture level	Compression rate (%)	Kyphosis (°) (Pre/Last)	Time to operation (months)	BMD (g/cm^2^)	Correction angle (°)	Main surgical indication
1	67	F	L_1_	45	16.7/10.4	5	0.582	6.3	Severe back pain d/t pseudarthrosis
2	71	F	L_1_	69	23.5/27.3	5	0.572	−3.8	Motor weakness
3	54	F	T_12_	64	22.8/18.3	4	0.691	4.5	Motor weakness, Hypesthesia
4	65	M	T_12_	63	24.5/6.2	5	0.711	18.3	Severe back pain d/t pseudarthrosis
5	75	F	T_9_	62	33.1/15.9	6	0.667	17.2	Gait disturbance
6	63	M	L_1_	53	20.7/10.9	3	0.732	9.8	Motor weakness
7	74	M	T_11_	70	17.6/8.9	4	0.714	8.7	Motor weakness, Hypesthesia
8	55	M	T_11_	65	30.2/29.2	2	0.712	1.0	Severe back pain d/t pseudarthrosis
9	78	M	L_1_	71	30.6/16.3	4	0.653	14.3	Severe back pain d/t kyphosis
10	59	M	T_12_	64	30.0/36.7	3	0.644	−6.7	Motor weakness, hypesthesia
11	79	F	T_12_	64	20.4/12.2	5	0.654	8.2	Gait disturbance
12	70	M	T_12_	69	15.3/15.1	1	0.705	0.2	Gait disturbance
13	52	F	T_12_	63	27.8/19.5	1	0.713	8.3	Severe back pain d/t pseudarthrosis
14	59	F	T_11_	83	15.6/14.7	4	0.652	0.9	Gait disturbance
15	65	M	T_12_	64	12.8/4.7	2	0.731	8.1	Motor weakness
16	68	F	T_11_	60	25.4/24.2	2	0.645	1.2	Gait disturbance
17	76	F	L_1_	65	29.5/16.7	2	0.571	12.8	Severe back pain d/t pseudarthrosis
18	77	M	T_12_	76	53.7/52.9	4	0.715	0.8	Severe back pain d/t kyphosis
19	55	F	L_1_	69	20.7/−0.9	6	0.740	21.6	Severe back pain d/t kyphosis
20	65	F	L_1_	64	52.1/19.7	4	0.661	32.4	Motor weakness
Average	66.4	—	—	65.15	26.2/17.9	3.6	0.673	8.2	—

BMD, Bone Mineral Density

### *Surgical Technique*

#### *Anesthesia and Position*

The patient was placed prone on bolsters on a radiolucent table. The table was flexed slightly initially so that returning to the horizontal plane would aid in closure of the osteotomy during surgery. The operative field was exposed using a posterior midline approach in the prone position with hypotensive anesthesia reducing the systolic blood pressure to 80–90 mm Hg.

#### *Approach and Exposure*

After posterior midline longitudinal skin incision, subcutaneous tissue and paraspinal muscles were subperiosteally dissected to expose lamina. Pedicle screws were inserted two levels above and below the osteotomy site.

#### *Modified Pedicle Subtraction Osteotomy*

**Figure 2:**
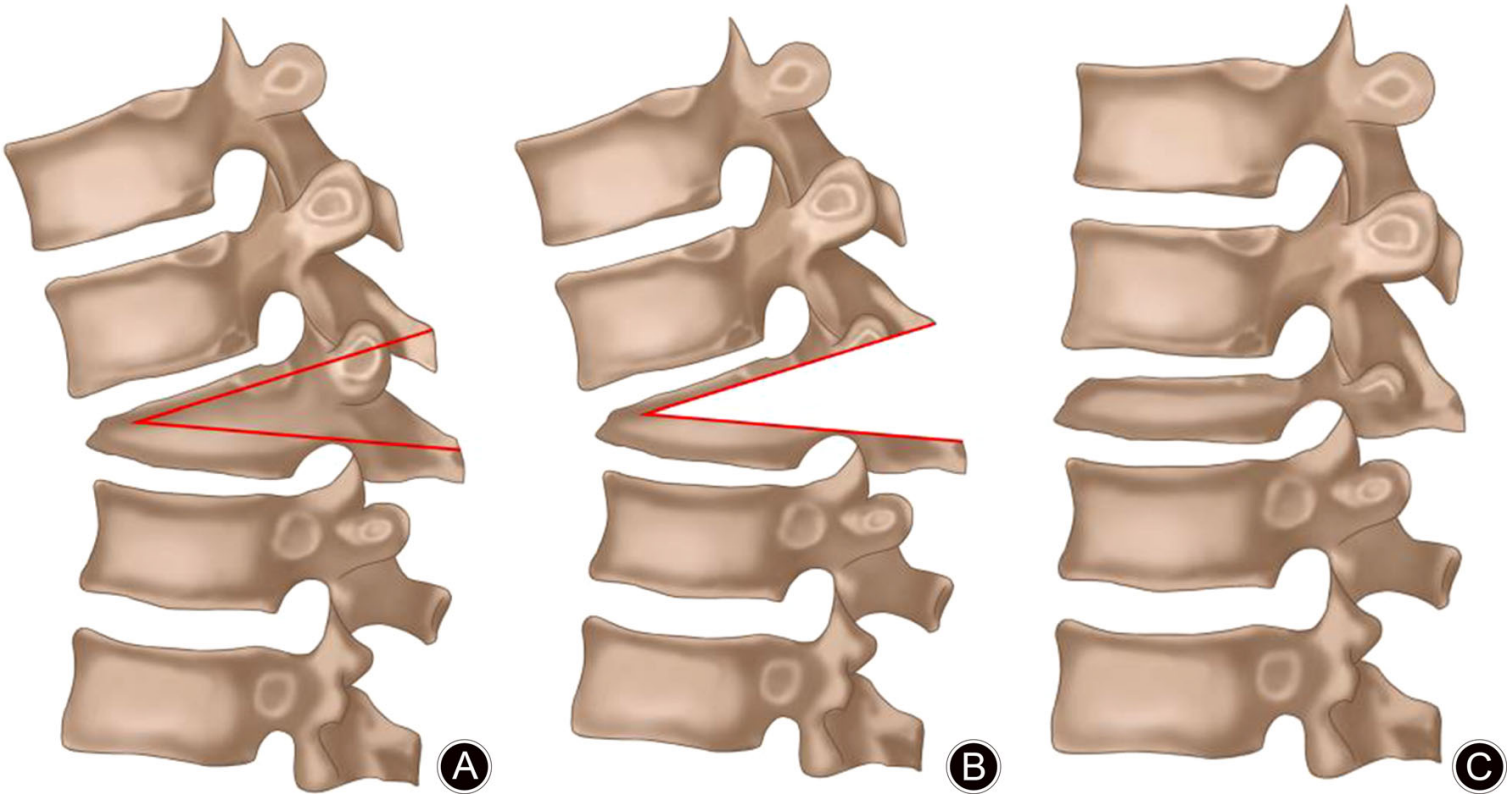
Schematic illustration of our osteotomy technique. (A) Planning of resection level before osteotomy. (B) After osteotomy. (C) Correction of kyphosis after osteotomy.

Our spinal osteotomy technique is a modification of the pedicle subtraction osteotomy that has the advantages of preserving the posterior neural arch, which reduces the development of epidural fibrosis and also provides a bed for fusion (Fig. 2). A vertebral body wedge osteotomy was performed just caudal to the superior endplate of the injured vertebrae^8^. The wedge was planned so that the apex was at the anterior one‐third of the vertebral body with the base located posteriorly.

The spinous process and lamina of the upper half of the injured vertebrae and the lower half of the superior adjacent vertebrae were excised to expose the thecal sac. The cephalad half of both pedicles of the injured vertebrae was then excised. Blunt dissection using gauze was used to expose the lateral cortex of the vertebral body. Using an osteotome, a first cut was made a few millimeters caudal and parallel to the superior endplate of the injured vertebrae up to the anterior one‐third of the body. A second cut was made 5–6 mm below and oblique to the first cut to mark out the wedge to be excised. The depth of the wedge and the direction of the cuts were confirmed fluoroscopically. The lateral cortex was then osteotomized up to the anterior one‐third of the body. The body was de‐cancellated to allow for closure of the osteotomy. If the upper end plate of the injured vertebral body was affected, the upper end plate and upper adjacent intervertebral disc were removed together.

A temporary stabilizing rod was placed to avoid sudden translation of the spinal column during the osteotomy. A similar procedure was performed on the contralateral side. Posterior fusion was performed from the two levels above to the two levels below using autologous bone grafts obtained from the body, lamina, and spinous process after osteotomy and instrumentation (Moss‐Miami, DePuy, Warsaw, IN, USA; Monarch, DePuy Spine, Inc., Raynham, MA, USA).

#### *Postoperative Management*

After surgery, the patient was ambulated in a thoracolumbosacral orthosis (TLSO) as soon as the pain alleviated and the brace was used for a period of 3 months. We tried to reduce the possibility of screw loosening by using screws that were 6–7 mm in diameter and 45–55 mm long, obtaining a favorable amount of initial kyphosis correction, and applying TLSO for 3 months.

### *Outcome Evaluation*

#### *Kyphotic Angle*

**Figure 3:**
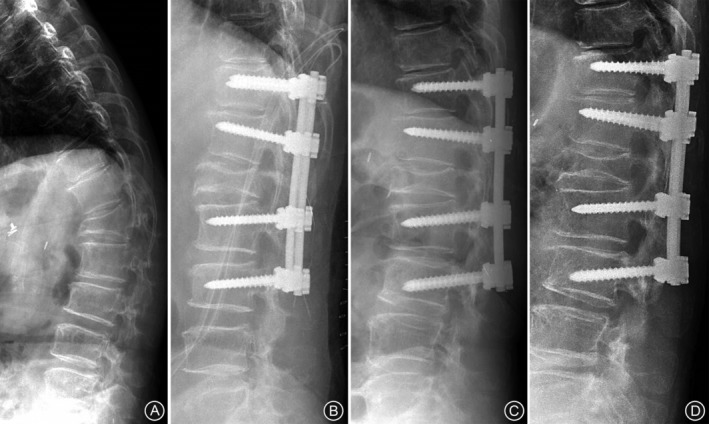
(A) Preoperative radiograph of a 65‐year‐old woman with an L_1_ osteoporotic compression fracture showing a kyphotic angle of 52.1 degrees. (B) Immediate postoperative radiograph showing kyphosis correction to 6.3 degrees. (C) The kyphosis progressed to 18.6 degrees at 2 years postoperative. (D) Lateral plain radiograph taken at 4 years postoperative showing well maintained sagittal correction with kyophosis of 19.7 degrees.

Kyphotic angle was measured using the Cobb angle method between the upper end plate of one level above and the lower end plate of one level below the injured vertebrae. Evaluation of the change in kyphotic angle was performed by comparing the preoperative, postoperative, and last follow‐up plain radiographs (Fig. 3).

#### *Japanese Orthopaedic Association (JOA) Score*

We evaluated the patient's clinical outcomes using Japanese Orthopaedic Association (JOA) scores. There were a total of 29 points from subjective symptoms (9 points), objective observations (6 points), restriction of activities of daily living (14 points), and urinary bladder function (‐6 points). Low JOA score was correlated with the degree of dysfunction.

#### *Oswestry Disability Index (ODI) Score*

We also evaluated Oswestry Disability Index (ODI) scores. The ODI questionnaire consists of 10 sections: pain, personal care, lifting, walking, sitting, standing, sleeping, sex life, social life, and traveling. Each question is scored from 0 (no dysfunction) to 5 (most obvious dysfunction). High ODI score was correlated with the degree of dysfunction.

#### *Patient Satisfaction*

The overall clinical patient satisfaction results were classified as poor, moderate, or excellent at the last follow‐up. A poor result was defined as when the patient's subjective symptoms did not improve or worsened with complications. A moderate result was defined as when the symptom improved less than 50%. An excellent result was defined as when the symptoms improved more than 50% compared to the preoperative status.

### *Statistical Analysis*

Descriptive statistics (arithmetic means, standard deviations, and ranges) were calculated using SPSS Windows ver. 14.0 (SPSS, Inc., Chicago, IL, USA). The paired *t*‐test was used to assess differences in the clinical and radiographic results before vs after surgery. *P* values < 0.05 were considered significant and the statistical analysis was independently performed by a statistician.

## Results

### *General Results*

Mean operation time was 4.2 h (range, 4–6 h). The overall mean EBL was 1098 mL (range, 750–1370 mL). All patients required postoperative blood transfusions. The mean blood transfusion was 1600 mL (range, 800–2800 mL). The mean hospital stay was 3.6 weeks (range, 2–6 weeks).

### *Kyphotic Angle*

The mean preoperative kyphotic angle was 26.2 ± 9.9 degrees (range, 12.8–53.7 degrees) and the mean postoperative kyphotic angle was 8.3 ± 8.1 degrees (range, −10.6 to 23.0 degrees), showing an 18.3‐degree correction with statistically significant differences (*P* < 0.01). Plain radiography at the last follow‐up showed a mean angle of 17.9 ± 12.6 degrees (range, −0.9 to 52.9 degrees), indicating a 9.5‐degree correction loss (*P* = 0.038). However, the overall correction was 8.2 degrees with statistically significant differences (*P* = 0.031), and successful bony union was achieved at the last follow‐up in all cases.

#### *Japanese Orthopaedic Association (JOA) Score*

All cases showed improvements in pain, and the mean JOA score significantly improved from preoperative 13.2 (range, 8–23) to last follow‐up 21.7 (range, 11–27) (*P* = 0.03).

#### *Oswestry Disability Index (ODI) Score*

The mean preoperative ODI score was 40.3 (range, 37–45), while the mean last follow‐up ODI score was 13.6 (range, 7–24) with statistically significant differences (*P* < 0.01).

#### *Patient Satisfaction*

Overall clinical patient satisfaction showed 12 excellent results (60%), five moderate results (25%), and three poor results (15%).

#### *Complications*

Ileus occurred immediately postoperatively in one case, but the patient recovered well with conservative treatment and early ambulation using the TLSO brace. There was one case of cauda equina syndrome at 2 days post‐operative. We performed emergency operation for hematoma removal and the motor grade recovered to fair. There were two cases of screw loosening, but in all cases the follow‐up plain radiographs showed a solid fusion and the screw loosening did not progress; therefore, revision surgery was not needed. There were no cases of distal junctional kyphosis, but there were two cases of proximal junctional kyphosis.

## Discussion

In general, OVCF results in kyphosis and local pain. It is usually not accompanied by neurological symptoms, and weak bone quality makes it difficult to insert implants during fracture surgery. Therefore, the treatment of OVCF in elderly patients is usually conservative, and these fracture heal well mostly without major complications^2^, ^9^, ^10^, ^11^, ^12^. However, despite sufficient conservative treatment, some patients suffer from severe pain and neurological symptoms due to deterioration of vertebral collapse and spinal instability^13^. In these cases, vertebroplasty or kyphoplasty may be the appropriate treatment option. However, vertebroplasty or kyphoplasty is less effective for spinal stability or neurological recovery, and Nakamae *et al*. also reported that it is ineffective in cases of high‐grade spinal canal compromise and an absence of remarkable dynamic instability^14^. Furthermore, increasing attention on health and developments in medicine have created rapid growth of the aging population. Therefore, the importance of active treatment, such as surgery, for OVCF is emphasized, especially in cases of neurological symptoms and/or spinal instability^4^, ^10^, ^11^, ^12^, ^15^, ^16^. Kaneda *et al*. reported the results for 22 patients who underwent surgery for OVCF. In particular, in cases of instability and kyphosis, neurological symptoms were common and the authors, therefore, emphasized the need for surgery^10^. In addition, Shikata *et al*. reported seven cases of compression fractures caused by osteoporosis that resulted in neurological impairment and recommended early surgical care for these cases whenever possible^4^. Various surgical methods, such as posterior decompression, posterior decompression and posterior fusion, anterior decompression and fusion with or without posterior fixation, and osteotomy, have been reported^4^, ^10^, ^11^, ^12^, ^15^, ^16^. In general, performing only posterior decompression for OVCF with neurological symptoms is not recommended since it can cause spinal instability and increase kyphosis, resulting in worsening of neurological symptoms. Since anterior reconstruction without internal fixation cannot provide sufficient fixation or withstand rotational forces and can cause re‐collapse or subsidence of bone graft into the vertebral body, anterior or posterior internal fixation in combination with anterior surgery is needed^4^, ^10^, ^11^, ^17^, ^18^. Salomon *et al*. performed anterior decompression after posterior instrumentation, but the long operation time and abdominal or thoracic open surgery were more likely to cause postoperative complications in elderly patients^19^. Ha *et al*. performed various surgical methods such as anterior fusion, posterior instrumentation, and anterior and posterior combined surgery for OVCF patients. They reported a preoperative kyphosis angle of 29.6 degrees, postoperative angle of 24.3 degrees, and last follow‐up angle of 29.3 degrees, resulting in a correction angle loss of 5 degrees. At the last follow‐up, the correction angle was 0.3 degrees, resulting in correction failure^15^.

Spinal osteotomy may decompress the spinal canal, restore the sagittal balance, and correct the kyphotic deformity. Although various techniques had been introduced by several authors since the Smith‐Peterson osteotomy was first attempted in patients with rheumatoid arthritis in 1945, this method has had many complications^20^. In 1984, Chewning *et al*. reported a surgical method called the egg‐shell procedure, which consists of removing the hemivertebra through the pedicle in congenital scoliosis patients. In 1985, Thomasen reported a procedure for removing the pedicle and the posterior trabecular bone without affecting the anterior column while shortening and correcting the middle and posterior columns. These procedures reduced the complications of the conventional osteotomy and were widely adopted^16^. Compared to anterior decompression after posterior instrumentation, posterior spinal osteotomy has the benefits of a single incision and avoiding abdominal or thoracic open surgery, resulting in additional postoperative complications. Suk *et al*. reported that a satisfactory therapeutic result was obtained by the posterior single approach compared with simultaneous anterior and posterior surgeries^21^. Okuda *et al*. reported that pedicle subtraction osteotomy can correct kyphosis^6^.

There are few reports of spinal osteotomy for the treatment of OVCF. However, in cases of pseudarthrosis due to healing failure of vertebral body fractures with kyphosis or in cases of neurological impairments, such as spinal cord compression or spinal stenosis, spinal osteotomy is a viable treatment option^22^. Specifically, the main advantages of our modified pedicle subtraction osteotomy are an early bone union and high bone union rate due to maximized bone contact surface and a favorable amount of kyphosis correction. Also, modified pedicle subtraction osteotomy reduces patient and surgeon stress as it uses the single posterior approach operation and enables early ambulation by applying orthosis after surgery. Rapid postoperative ambulation decreases the possibility of postoperative complications such as thromboembolic events^23^, ^24^. However, the high bleeding risk and difficult surgical technique limit the general application of spinal osteotomy for OVCF. In particular, elderly patients had a higher rate of major postoperative complications, which were defined as life‐threatening and requiring reoperation^25^. Increased age, comorbidities, blood loss, operative time, and number of levels are the risk factors for these complications^26^, ^27^, ^28^. Due to these causes, spinal osteotomy cannot be performed in all OVCF patients. As three of our 20 cases involved complications, full sagittal correction of OVCF through spinal osteotomy to restore the sagittal balance and correct the kyphotic deformity should be carefully considered. We performed a single‐level osteotomy in all patients. We planned not to perform full correction considering the other complications. Therefore, the mean initial correction angle of kyphosis was 18 degrees in our study, which was not larger than the correction angle of the pedicle subtraction osteotomy. In our study, the mean preoperative kyphosis was 26.2 degrees and mean immediate postoperative kyphosis angle was 8.3 degrees, resulting in an 18.3‐degree correction effect. At the last follow‐up, the mean kyphosis angle was 17.9 degrees, showing a 9.5‐degree correction loss. Our last correction angle was 8.2 degrees, a moderate value compared to previous other studies. To prevent adjacent segmental pathology and minimize fusion levels, we extended the fusion above and below by two levels compared to conventional osteotomy, which features three‐level fusion above and below. This may result in more correction loss in the follow‐up data. Also, there were two cases in our study with screw loosening affecting correction loss larger than the mean.

The limitations of this study are its retrospective design, relatively small number of patients, and lack of a control group treated with other methods. However, the surgery was performed in a single institution by one surgeon with the limited indications mentioned above. Thus, there is a limit to our study case. In future studies, we will expand the number of cases to reinforce our results.

The purpose of our treatment for OVCF was to prevent the progression of kyphotic deformity, relieve pain, and allow the patient to return to their normal activities, not to achieve normal sagittal balance. However, some extent of sagittal alignment correction should be obtained to avoid adjacent segment pathology or additional collapse. There were reported cases of OVCF in which sagittal alignment correction was obtained with other simpler methods, but spinal osteotomy was sometimes needed. Therefore, we tried to correct a limited degree of sagittal imbalance through osteotomy. In conclusion, for the treatment of OVCF, the goals of our modified pedicle subtraction osteotomy were to improve severe pain and neurological impairments resulting from pseudarthrosis and kyphotic deformity. This modified pedicle subtraction osteotomy technique can achieve early and high bone union and obtain a favorable amount of kyphosis correction. It also has the advantage of needing a single procedure that requires no anterior surgery and early ambulation with orthosis after surgery. However, the high bleeding risk, difficult surgical technique, and possibility of neurological damage are limitations of spinal osteotomy. We do not think that our osteotomy technique is appropriate for the treatment of every case of OVCF. However, this modified pedicle subtraction osteotomy technique may be an appropriate surgical intervention for indicated cases of OVCF.

## Disclosure

This research was supported by the Basic Science Research Program through the National Research Foundation of Korea (NRF) funded by the Ministry of Education (NRF‐2018R1D1A1A02086142) and a grant (CRI 17016‐1) from the Chonnam National University Hospital Biomedical Research Institute.
